# Reliability and Validity of the Czech Version of the Pittsburgh Sleep Quality Index in Patients with Sleep Disorders and Healthy Controls

**DOI:** 10.1155/2021/5576348

**Published:** 2021-08-10

**Authors:** Denisa Manková, Daniela Dudysová, Jan Novák, Eva Fárková, Karolina Janků, Monika Kliková, Jitka Bušková, Aleš Bartoš, Karel Šonka, Jana Kopřivová

**Affiliations:** ^1^National Institute of Mental Health, Topolová, 25067 Klecany, Czech Republic; ^2^Third Faculty of Medicine, Charles University, Ruská 87, 100 00 Prague, Czech Republic; ^3^Department of Anthropology and Human Genetics, Faculty of Science, Charles University, Viničná 7 128 00 Prague, Czech Republic; ^4^Department of Neurology and Center of Clinical Neuroscience, Charles University, First Faculty of Medicine and General University Hospital, Kateřinská 30, 128 21 Prague, Czech Republic

## Abstract

**Objectives:**

Psychometric properties of the Czech version of the Pittsburgh Sleep Quality Index (PSQI-CZ) have been evaluated only in patients with chronic insomnia, and thus, it is unclear whether PSQI-CZ is suitable for use in other clinical and nonclinical populations. This study was aimed at examining the validity and reliability of the PSQI-CZ and at assessing whether the unidimensional or multidimensional scoring of the instrument would be recommended.

**Methods:**

A total of 524 adult subjects from the Czech population participated in the study. The internal consistency of PSQI was evaluated using Cronbach's alpha. The known-group validity was tested using the Kruskal-Wallis *H* test to verify the difference between patients with sleep disorders and healthy control sample. For testing the structural validity, a cross-validation approach was used with both exploratory factor analysis (EFA) and confirmatory factor analysis (CFA). For EFA, the maximum likelihood method with direct oblimin rotation and parallel analysis was used.

**Results:**

The internal consistency of PSQI-CZ items was moderate (*α* = 0.75). Receiver operating characteristic (ROC) curve analysis showed high specificity (0.79) and moderate sensitivity (0.64) using an optimal cut-off score of 10. The EFA revealed a 3-factor structure with factors labelled as “sleep duration and efficiency,” “sleep disturbances and quality,” and “sleep latency.” The CFA showed that the emerged 3-factor model had a partly acceptable fit, which was better than other previously supported models.

**Conclusions:**

A high cut-off score of 10 is recommended to define poor sleep quality. Given the inconsistency of structural analyses, alternative scoring was not recommended. However, the individual components in addition to a total score should be interpreted when assessing sleep quality. We recommend editing and verifying the PSQI-CZ translation.

## 1. Introduction

Disturbed sleep represents one of the most frequent health issues. It has been shown that more than half of the adult population of economically developed countries experience unpleasant sleep disturbance [1]. The functioning of the sleep cycle can be verified by objective methods such as polysomnography or actigraphy. However, when assessing sleep, it is important to take into account the subjectively perceived quality of sleep as well as other variables such as comorbidities and environment. If we look at the quality of sleep by objective methods, sleep quality can involve several different parameters including sleep onset latency, sleep duration, sleep efficiency, and a number of awakenings [2]. Disruption, abnormality, or irregularity of some of these measures leads to a decrease in sleep quality. The prevalence of symptoms of difficulty in initiating or maintaining sleep ranges from 10% to 48% in the general population [3]. Poor sleep quality can contribute to absence from work, accidents at the workplace, and increased risk of negative health consequences such as sleep and neuropsychiatric disorders [4].

Although polysomnography is considered the gold standard for measuring sleep quality, the Pittsburgh sleep quality index (PSQI) is the most commonly used subjective measure that assesses important aspects of the sleep quality and the presence of symptoms of frequent sleep disorders in both clinical and research settings (see more in Section 2.2.). The PSQI has been translated into more than 46 languages. All language versions are managed by Mapi Research Trust and are available subject to compliance with the prescribed conditions of use (research, clinical practice). It is unknown whether the Czech version of PSQI officially distributed by Mapi Research Trust is an appropriate translation of the instrument. As stated on the PSQI distributors' website (http://eprovide.mapi-trust.org), the listed translations may not have undergone a full linguistic validation process and may require further clarification. Nevertheless, studies of different language versions have demonstrated a good internal consistency (Cronbach's alpha coefficient ranging from 0.71 to 0.85) and appropriateness of using the PSQI in clinical and population studies [3, 5–10].

Validity and reliability of the PSQI have been verified by comparisons of healthy control groups with clinical populations of patients with psychiatric disorders [11, 12], sleep disorders [8, 9, 13], or somatic disorders [14, 15]. Although studies have shown good validity and reliability of the questionnaire across a different spectrum of research groups, there is no uniform concept of its structural validity. A recent review pointed out that most structural validation studies had some shortcomings (e.g., inappropriate sample, unused Kaiser-Meyer-Olkin test, Bartlett's test of sphericity, and lacking one of the factor analysis approaches or its relevant details). Insufficient or incorrectly chosen statistical methods may then create doubts about the described factor structures in individual research samples [16]. There are currently three most common model proposals. The original single-factor model suggests that a single summed total score best captures the multidimensional nature of sleep disturbance as indexed by the PSQI [11, 12]. The original single-factor model was confirmed by several studies [17, 18]. Other models question Buysse et al.'s combination of all seven PSQI components into one factor. Some suggest using 2-factor models (e.g., [5, 6, 14, 19, 20, 21]). One of the more replicated models proceeds from a study by Magee et al. [19], who suggested the following factors: (1) sleep efficiency—based on the values of two components sleep duration and habitual sleep efficiency and (2) perceived sleep quality—based on subjective sleep quality, sleep latency, sleep disturbance, use of sleep medications, and daytime dysfunction [19, 22]. Other studies copy Magee et al.'s model to the exclusion of the use of sleep medication component [20, 21]. Others recommend a 3-factor structure, which is based on Cole et al.'s study [12, 23, 24]. Cole et al. proposed three factors: (1) sleep efficiency (based on sleep duration, habitual sleep efficiency), (2) perceived sleep quality (based on subjective sleep quality, sleep latency, and use of sleep medications), and (3) daily disturbances (based on sleep disturbances and daytime dysfunction) [12]. Although no consensus has been reached, the original unidimensional scoring system and further validation were more recently recommended [1, 16].

Although the PSQI is widely used in research and clinical practice in the Czech Republic, psychometric characteristics of its Czech version (PSQI-CZ) have been evaluated only in patients with chronic insomnia [25]. Thus, the study was aimed at examining the known-group and construct validity and reliability (internal consistency) of the PSQI-CZ and at assessing whether the unidimensional or multidimensional scoring of the instrument would be recommended.

## 2. Materials and Methods

### 2.1. Study Sample

Data was collected at three clinical and research sites: Department of Neurology, First Faculty of Medicine, Charles University; Department of Sleep Medicine, National Institute of Mental Health; and private neurological clinic INSPAMED. Participants were recruited as part of 3 studies: a longitudinal study on aging and memory, the insomnia treatment programme at the National Institute of Mental Health (Czechia, NIMH-CZ), and a study directly focused on validation of the PSQI-CZ. The local institutional review boards approved the study (Ethics Committee of the General University Hospital Prague, No. 1774/15D; Ethics Committee of NIMH-CZ, No. 170/16). The study protocol was in line with international ethical standards [26]. All subjects were examined with the Czech version of PSQI, which was distributed by Mapi Research Trust. Basic sociodemographic information (age, sex, and diagnosis) has also been obtained. Answers were filled out in a paper-and-pencil form among the general population and people with sleep disorders between 2015 and 2018. In the patient group, the diagnostic categories were determined according to ICD-10. The native language of all participants was Czech. We had the data available from a total of 583 adults. We then excluded individuals under 18 and above 80 years old. An incompletely or incorrectly filled out questionnaire was the second exclusion criterion. Finally, we excluded patients with the unspecific or combined diagnoses. In total, 59 subjects were excluded. We did not perform any multiple imputations to address the missing values. From the remaining 524 adult probands who were included in the study, 326 probands were sleep laboratory patients (patients with sleep disorders (SDis)); the remaining 198 subjects formed the control group (HC). The HC group consisted of volunteers from the Czech population who responded to the invitation to participate in the research and stated that they do not suffer from any sleep and psychiatric disorder while other somatic disorders were not monitored.

### 2.2. PSQI

The PSQI was developed by Buysse et al. in 1989. It measures the quantitative and subjective aspects of sleep quality. The PSQI consists of 19 self-rated items and seven clinically derived domains of sleep difficulties in the past month: subjective sleep quality, sleep latency, sleep duration, habitual sleep efficiency, sleep disturbances, use of sleep medication, and daytime dysfunction. Each of these domains is weighted equally on a 0-3 scale. The seven component scores are summed to yield the total global PSQI score, which ranges between 0 and 21 points. A total PSQI score > 5 denotes worse sleep quality [11], although some studies recommend that a higher cut-off score of 6 [8, 9], 7 [18, 27], 8 [15], or even 8.5 [13] would increase the PSQI's specificity and lead to a very small decrease in its sensitivity. The questionnaire also consists of 5 additional questions that are rated by a bed partner or a roommate. The latter five questions are used for clinical information only [11].

### 2.3. Statistical Analysis

PSQI scores were not normally distributed both in the control and patient samples (Shapiro − Wilk < 0.01). The known-group validity was tested using the Kruskal-Wallis *H* test for the confirmation of the presence of the difference between patient and control samples. The effect size was calculated using eta squared (*ε*^2^) and evaluated using following criteria: 0.01-<0.06 (as small effect), 0.06-<0.14 (as moderate effect), and ≥0.14 (as large effect). The test characteristics and an optimal cut-off score were calculated and tested using the receiver operating characteristic (ROC) curve [28]; the optimal cut-off value was estimated using two methods: by the position closest to the top-left corner of the curve and by using the maximum value of Youden index [29].

The internal consistency of the PSQI was tested with Cronbach's alpha [30]. A reliability statistic of 0.70 was considered acceptable, a range between 0.70 and 0.60 was questionable, and values lower than 0.60 were considered inadequate for the internally consistent instrument [31, 32]. Independence on factors (age and sex) was tested using basic linear models.

For testing the factor structure, a cross-validation approach was used; i.e., the study sample was randomly divided into two adequately sized subsamples; the first subsample was used for factor identification using exploratory factor analysis (EFA). The Bartlett test of sphericity and Kaiser-Meyer-Olkin test were used for verifying the suitability for the analysis. We used the following criteria for factor extraction: eigenvalues > 1, loadings of items ≥ 0.35 [33], and all selected factors from the real data had to perform better in eigenvalue than factors from the random data. The maximum likelihood method with direct oblimin rotation was used for factor extraction, as we assumed correlation between components. The number of factors retained was estimated using parallel analysis, i.e., a data-driven approach comparing the observed eigenvalues of a correlation matrix with those from the random data [34].

The second subsample was then used for testing the emerged model and compare the goodness of fit with other published models using confirmatory factor analysis (CFA). Our proposed model was compared with previously published and supported models: the original 1-factor model [11], the 3-factor model first published by Cole et al. [12], and the 2-factor model first published by Magee et al. [19]. To assess model fit, multiple fit indices were used and considered good: comparative fit index (CFI) at ≥0.95 (or ≥0.90 for acceptable fit), Tucker-Lewis index (TLI) at ≥0.95 (or ≥0.90 for acceptable fit), standardized root mean square residual (SRMR) at ≤0.08, and root mean square error of approximation (RMSEA) at ≤0.05 (or ≤0.08 for adequate fit) along with 90% confidence intervals (90% CI). Statistically nonsignificant and lower chi-squared tests (*χ*^2^) were also considered to identify better models [35]. To determine the best model which fits our data, all models were compared to each other using Bayesian information criterion (BIC), *χ*^2^ difference tests (Δ *χ*^2^), and RMSEA CI overlap. As in Cole et al. [12], a model was considered better fitted if at least two of the three criteria for significant differences were met; i.e., it had a lower BIC (by at least 10 points), lower nonoverlapping RMSEA CIs, and a significantly different Δ *χ*^2^ where a model with lower *χ*^2^ was better.

The whole analysis was performed in R language version 3.5.1 [36] and jamovi version 1.1 [37]; the following packages in R were used: Tidyverse group of packages [38], psych [39], cutpointr [40], and pROC package [41].

## 3. Results

### 3.1. Descriptive Statistics of the Studied Sample

The details of the subscale scores and total PSQI scores in our subsamples and whole sample are displayed in Table 1. The sleep disorder group included 196 women and 130 men (1.51 woman to man ratio, the significant difference observed, Kruskal-Wallis *χ*^2^ = 8.06, *p* < 0.01, *ε*^2^ = 0.02) as opposed to the control group which includes 128 women and 70 men (1.83 women to man ratio, the nonsignificant difference observed, Kruskal-Wallis *χ*^2^ = 0.76, *p* < 0.38, *ε*^2^ = 0.00). The primary condition of most patients was insomnia (*n* = 202), followed by obstructive sleep apnea (*n* = 58), somnambulism (*n* = 19), hypersomnia (*n* = 19), narcolepsy and cataplexy (*n* = 13), nightmare disorder (*n* = 4), REM sleep behaviour disorder (*n* = 3), restless legs syndrome (*n* = 3), sleep terrors (*n* = 3), and circadian rhythm sleep disorder (*n* = 2).

### 3.2. Reliability: Internal Consistency

We tested the reliability of the PSQI-CZ by estimation of PSQI-CZ internal item consistency using Cronbach's alpha coefficient. The overall internal consistency of PSQI-CZ items was adequate (*α* = 0.75). Dropping any of the components did not result in a higher internal consistency (Table 2). The internal consistency of the PSQI was higher among patients (*α* = 0.71) than controls (*α* = 0.63).

All PSQI components were positively correlated with the PSQI total score. The largest component-to-total-score correlation was observed for sleep duration (*r* = 0.74, *p* < 0.001) and subjective sleep quality (*r* = 0.73, *p* < 0.001), the lowest for habitual sleep efficiency (*r* = 0.54, *p* < 0.001) and sleep latency (*r* = 0.55, *p* < 0.001). The largest observed component-to-component correlation was observed between subjective sleep quality and sleep disturbance (*r* = 0.59, *p* < 0.001), and the lowest between habitual sleep efficiency and sleep disturbance (*r* = 0.07, *p* < 0.001).

### 3.3. Validity

We tested known-group validity on a sample of healthy controls (HC) and patients with a diagnosed sleep disorder (SDis). The patient group had a higher global score of PSQI-CZ (11.53 ± 4.51) in comparison with the mean PSQI value of the control group (6.56 ± 3.24); the difference was significant and relevant (Kruskal-Wallis *χ*^2^ = 131.22, *p* < 0.001, *ε*^2^ = 0.25), with an average mean difference of 4 points.

**Figure 1:**
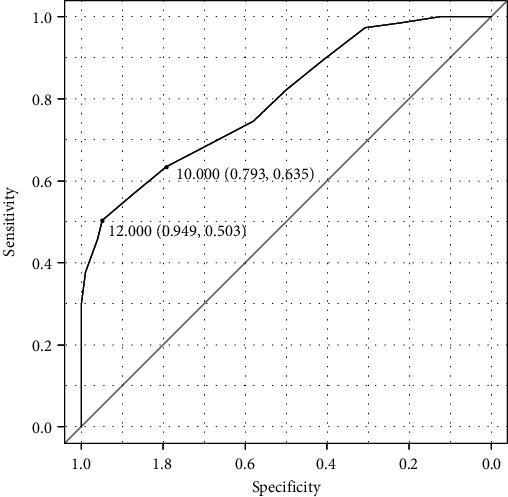
ROC curve for optimal PSQI cut-off values selected by the highest Youden index and position closest to the top-left corner of the curve. The sensitivity and specificity for the cut-off values of 12 identified with the highest Youden index and 10 identified by its position closest to the top-left corner of the curve are shown.

ROC analysis showed high specificity (0.79) and low sensitivity (0.635) using a cut-off score of 10 specified as a point closest to the top-left corner of the curve. Using the identification of cut-off value using the maximum Youden index, the optimal cut-off value was 12 with very high specificity (0.94) and very low sensitivity (0.50). The original recommended cut-off score of 5 was highly unspecific (Table 3, Figure 1). The total area under the curve (AUC) was 0.80.

### 3.4. Exploratory Factor Analysis

We tested structural validity using a cross-validation approach with both exploratory factor analysis (EFA) and confirmatory factor analysis (CFA). Prior to analysis, PSQI components were tested for sphericity using Bartlett's test (*χ*^2^ = 464.45, *p* < 0.01) and sampling adequacy with the Kaiser-Meyer-Olkin test (KMO = 0.72). It was thus appropriate to proceed with EFA. Using EFA, a 3-factor model was identified (Table 4) using data-driven parallel analysis with the sum of the squared loadings (eigenvalues) 1.38, 1.38, and 1.10. The first factor explaining 19.85% of variance was termed sleep duration and efficiency with the highest loading in sleep duration. The second factor was termed sleep disturbances and quality with the highest loading in sleep disturbances followed by subjective sleep quality and daytime dysfunction components. The third factor was labelled as sleep latency and was loaded by sleep latency and sleep medication use components. All components were included as none of the loadings reached the minimum critical value of 0.35. The whole model was able to describe 55.11% of the variability. The correlations between factors were moderate (*r* = 0.33-0.54) [42].

### 3.5. Confirmatory Factor Analysis

**Figure 2:**
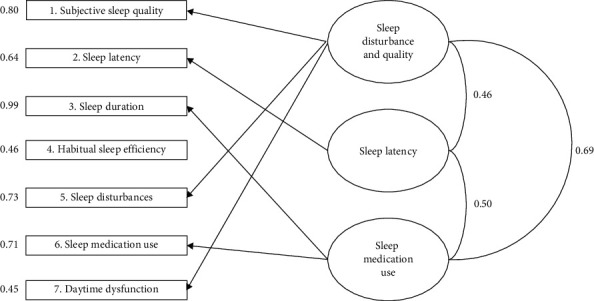
Confirmatory factor analysis (CFA) of our 3-factor solution of the PSQI. Ovals represent factors; rectangles represent seven components of the sleep quality subscales. Numbers next to rectangles denote standardized path coefficients, whereas numbers next to the factors represent factor correlations.

To cross-validate our 3-factor solution, the second half of our test sample was used for CFA. CFA was also performed on the original 1-factor model [11] and two established 3- and 2-factor models as in Cole et al. [12] and Magee et al. [19], respectively. The goodness-of-fit indices for all selected models were performed and are shown in Table 5. The goodness-of-fit statistics for our proposed 3-factor solution and for Cole et al.'s model was acceptable for CFI and SRMR while other indices were insufficient. Both Buysse et al.'s and Magee et al.'s models were insufficient in all indices except the SRMR. When our model was compared to other models, our 3-factor solution was significantly better fitted than all other models. Descriptively, we also found that both Cole et al.'s and Magee et al.'s models were better fitted than Buysse et al.'s original model and that Cole et al.'s model was not better fitted than Magee et al.'s model. Loadings in our CFA model were adequate, ranging from good to excellent (0.45 to 0.99). The correlations between factors were 0.46, 0.50, and 0.69 (medium effect) (Figure 2).

## 4. Discussion

To the best of the author's knowledge, this is the first study to examine the psychometric characteristics of the Czech version of the PSQI in various study samples (patients with sleep disorders and healthy volunteers). Our results demonstrate that the global internal consistency of the PSQI-CZ is lower (*α* = 0.75) than in the original study (*α* = 0.83) [11]. Given the characteristics of our sample, studies working with patients with sleep disorders show both similar [18], lower [25], and higher values of internal consistency [8, 9, 13]. Our Cronbach`s alpha was thus adequate and comparable to other studies that recommend the use of the questionnaire in clinical practice and research. Similar levels of Cronbach's alpha can be found in studies performed in psychiatric patients [7, 43], cancer patients [14], the general healthy population [3, 24], and adolescents [5, 44].

In contrast to other previously published studies [5, 6, 13, 18, 27], dropping any of the PSQI components did not result in a higher internal consistency in our research sample. Similarly and in contrast to our findings, some studies tend to exclude one or more PSQI components (e.g., daytime dysfunction, sleep medications use) as a result of factor analyses [5, 6, 20, 21, 27, 45–47]. Our findings however allowed for keeping all components, which was also shown in previous studies [11, 12, 19, 22–24, 48]. The differences in results may be attributed to diversity in sample characteristics. Our study included the general healthy population as well as patients with sleep disorders, which is in contrast to other studies validating PSQI in specific populations such as centenarians [27], adolescents [5], pregnant women [6], and psychiatric patients [7, 43].

The PSQI factor structure is a controversial research topic as the widely used original one-factor model may not be satisfactory in all populations. In the present study, we used a cross-validation approach using the first EFA and a series of CFAs including the most published structures [11, 12, 19]. The results of our factor analyses did not show entirely consistent results. Our exploratory factor analysis revealed the same 3-factor structure as in the Peruvian sample of college students in Gelaye et al.'s study [22]. Our structure was different from the original 1-factor structure [11] and other commonly proposed structures [12, 19]. The 3-factor model in Peru explained approximately 59% of the total variance [22], and ours comparably 55% of the variability. A confirmatory factor analysis verified our emerged structure but showed only a partly acceptable fit for our model. We found a similarly acceptable fit for a model from Cole et al. [12]. However, when we compared Cole et al.'s model with our model, our model resulted in a significantly better fit. Present findings thus do not confirm previously found support for Cole et al.'s structure in a Czech insomnia sample [25]. The discrepancy with other studies can be attributed to differences in studied populations, diverse sample characteristics, nonuniform methodologies (e.g., factor rotation and extraction methods, estimation method selection) and highlights the inconsistency of structural validity of the PSQI across varied clinical and nonclinical populations [1, 27].

Together, our data point to limited usability of changing the factor structure or developing alternative scoring of the instrument. Based on the present findings, it is recommended that somnologists and other professionals should not solely rely on the overall PSQI score describing sleep quality. Instead, they ought to look at all components or at least at the components with consistently high loadings (i.e., sleep duration, subjective sleep quality, and sleep disturbances).

In line with other studies [8, 13], our results showed that the patient group had a significantly higher total score of PSQI-CZ than general controls. The difference between these groups was confirmed by large effect size. Our findings point to an unexpected result of a high value of 10 for an optimal cut-off score, respectively, 12 using the maximum Youden index value criterion. We recommend using a cut-off score of 10 based on its clinical relevance, i.e., the best ratio between sensitivity (0.64) and specificity (0.79) in comparison to score 12 based on the Youden index with high specificity (0.95) but mediocre sensitivity (0.50). The traditional cut-off score (>5) has previously been reported to be insufficient to distinguish between healthy and diseased subjects, and higher cut-off scores have been proposed [13, 15, 49]. However, to the authors' knowledge, no other study proposed such a high cut-off score. Gomes et al. published that the optimal cut-off of 5 was to detect self-reported poor/good sleepers in nonclinical settings. To discriminate nonclinical from clinical sleep patients, the optimal cut-off was >7 [18, 27]. Given the high average total PSQI score in our HC group, it is thus possible that the group included individuals who had undiagnosed or untreated sleep disorders. The absence of the disease does not mean that the person sleeps well and, conversely, that the patient with a certain diagnosis sleeps subjectively poorly [50]. Moreover, it can be assumed that people who entered the study as healthy controls may have a greater degree of self-observation and interest in health. A higher level of self-observation of various changes, differences, and symptoms can then reflect a higher score in the PSQI. High values in the overall PSQI score can be explained, especially for young adults, also by the influence of social factors such as demands during university studies [51], loneliness [52], interest in sports activities [53], or the action of blue light when using electronic devices [54].

Our study had several limitations. Firstly, the results of the correlations suggest that there may be a translation discrepancy in question number one for PSQI-CZ. Respondents might have mistaken the meaning of going to bed (lying down) with falling asleep when answering the first question of the PSQI-CZ. It would be worthwhile to make a linguistic adjustment of the Czech version and verify whether it changes the psychometric outcomes of the PSQI. Secondly, as subjects in our control group were considered healthy based on their self-assessment, the potential inclusion of persons with undiagnosed sleep disorders in the control group is a further limitation of our study. Nevertheless, we consider the findings important for three reasons. Primarily, our study is the first that mapped the statistical properties of the Czech version of the PSQI on a relatively large research sample which included both healthy controls and patients with sleep disorders. Secondly, the higher cut-off found for this translation is an important information for clinical practice. And finally, our data demonstrated a 3-factor structure of the Czech PSQI that was not found useful for establishing an alternative scoring system.

## 5. Conclusion

For the current official Czech translation of the PSQI, a cut-off score higher than 10 is recommended to define poor sleep quality. Furthermore, not only the total score but also the results of the individual components should be taken into account. It is suggested that PSQI-CZ with a modified question should be created to verify respondents' understanding of the meaning of questions. Further studies on the psychometric properties of PSQI-CZ in various research samples (e.g., general population, somatic disorders) including the test-retest reliability and verification of a modified translated version would strengthen our understanding of the potential benefits and limitations of PSQI-CZ in clinical and research practice in the Czech Republic.

## Figures and Tables

**Table 1:** The mean of PSQI subscales and PSQI total scores in the control group, patient group, and whole sample.

PSQI components		Controls (HC)		Patients (SDis)		Whole sample	
		Mean	SD	Mean	SD	Mean	SD
PSQI_1	Subjective sleep quality	0.91	0.74	1.56	1.14	1.31	1.05
PSQI_2	Sleep latency	0.83	0.79	1.71	0.7	1.38	0.85
PSQI_3	Sleep duration	0.95	0.98	1.68	1.12	1.40	1.12
PSQI_4	Habitual sleep efficiency	1.2	1.25	1.97	0.96	1.68	1.14
PSQI_5	Sleep disturbance	1.26	0.53	1.38	1.16	1.34	1.98
PSQI_6	Use of sleep medications	0.17	0.52	1.85	0.95	1.21	1.15
PSQI_7	Daytime dysfunction	1.24	0.79	1.38	1.31	1.33	1.14
PSQI_total		6.56	3.24	11.53	4.51	9.65	4.73

SD: standard deviation.

**Table 2:** Item reliability statistics. Item-rest correlation and Cronbach's *α* if this item is dropped for each PSQI component in the whole sample (combined HC and SDis groups) are shown.

		Cronbach's *α* if item dropped
PSQI_1	Subjective sleep quality	0.69
PSQI_2	Sleep latency	0.73
PSQI_3	Sleep duration	0.69
PSQI_4	Habitual sleep efficiency	0.75
PSQI_5	Sleep disturbance	0.71
PSQI_6	Use of sleep medications	0.72
PSQI_7	Daytime dysfunction	0.74

**Table 3:** Different cut-off values selected by the highest Youden index. The sensitivity (%), specificity (%), positive likelihood ratio (LR+), negative likelihood ratio (LR−), and calculated Youden index for specific cut-off values are shown. Values were selected by the highest Youden index (9, 10, 11, 12, and 13) and compared with a value (5) recommended in the original work by Buysse et al. [11]. The total area under curve (AUC) was 0.80.

Cut-off point	Sensitivity (%)	Specificity (%)	LR+	LR−	Youden index
5	97.24%	30.81%	1.41	0.09	0.28
9	68.71%	69.19%	2.23	0.45	0.38
10	63.5%	79.29%	3.07	0.46	0.43
11	56.75%	87.37%	4.49	0.50	0.44
12	50.31%	94.95%	9.96	0.52	0.45
13	45.71%	95.96%	11.31	0.57	0.42

**Table 4:** Exploratory factor analysis for the 3-factor solution of the PSQI-CZ. Factor analysis conducted using the maximum likelihood extraction method and oblimin rotation.

PSQI component	Sleep duration and efficiency	Sleep disturbances and quality	Sleep latency
Subjective sleep quality		0.63	
Sleep latency			0.73
Sleep duration	0.99		
Habitual sleep efficiency	0.45		
Sleep disturbances		0.84	
Sleep medication use			0.60
Daytime dysfunction		0.35	
Variance explained	19.85%	19.54%	15.72%

**Table 5:** Goodness-of-fit indices for selected models.

	*χ* ^2^	df	*p* value	CFI	TLI	SRMR	RMSEA	RMSEA 90% CI		BIC
Our model	41.73	11	<0.001	0.93	0.86	0.05	0.10	0.07	0.14	5033
Buysse et al. [11]	99.27	14	<0 .001	0.80	0.70	0.07	0.15	0.13	0.18	5074
Cole et al. [12]	52.26	11	<0.001	0.90	0.82	0.06	0.12	0.09	0.15	5044
Magee et al. [19]	62.66	13	<0.001	0.88	0.81	0.06	0.12	0.09	0.15	5043

*χ*^2^: chi-squared statistic; df: degrees of freedom; CFI: comparative fit index; TLI: Tucker-Lewis index; SRMR: standardized root mean square residual; RMSEA: root mean square error of approximation; RMSEA 90% CI: 90% confidence interval of the RMSEA; BIC: Bayesian information criterion.

## Data Availability

The data used to support the findings of this study are available from the corresponding author upon request.

## References

[1] Mollayeva T., Thurairajah P., Burton K., Mollayeva S., Shapiro C. M., Colantinio A. (2016). The Pittsburgh sleep quality index as a screening tool for sleep dysfunction in clinical and non-clinical samples: a systematic review and meta-analysis. *Sleep Medicine Reviews*.

[2] Krystal A. D., Edinger J. D. (2008). Measuring sleep quality. *Sleep Medicine*.

[3] Hinz A., Glaesmer H., Brähler E. (2017). Sleep quality in the general population: psychometric properties of the Pittsburgh Sleep Quality Index, derived from a German community sample of 9284 people. *Sleep Medicine*.

[4] Buysse D. J., Germain A., Moul D., Nofzinger E. A., Buysse D. J. (2005). Insomnia. *Sleep Disorders and Psychiatry*.

[5] Passos M. H. P., Silva H. A., Pitangui A. C. R., Oliveira V. M. A., Lima A. S., Araújo R. C. (2017). Confiabilidade e validade da versao brasileira do Índice de Qualidade do Sono de Pittsburgh em adolescentes. *Jornal de Pediatra*.

[6] Qui C., Gelaye B., Zhong Q.-Y., Enquobahrie D. A., Frederick I. O., Williams M. A. (2016). Construct validity and factor structure of the Pittsburgh Sleep Quality Index among pregnant women in a Pacific-Northwest cohort. *Sleep & Breathing*.

[7] Doi Y., Minowa M., Uchiyama M. (2000). Psychometric assessment of subjective sleep quality using the Japanese version of the Pittsburgh Sleep Quality Index (PSQI-J) in psychiatric disordered and control subjects. *Psychiatry Research*.

[8] Backhaus J., Junghanns K., Broocks A., Riemann D., Hohagen F. (2002). Test-retest reliability and validity of the Pittsburgh Sleep Quality Index in primary insomnia. *Journal of Psychosomatic Research*.

[9] Tsai P.-S., Wang S.-Y., Wang M.-Y. (2005). Psychometric evaluation of the Chinese version of the Pittsburgh Sleep Quality Index (CPSQI) in primary insomnia and control subjects. *Quality of Life Research*.

[10] Takács J., Bódizs R., Ujma P. P., Horváth K., Rajna P., Harmat L. (2016). Reliability and validity of the Hungarian version of the Pittsburgh Sleep Quality Index (PSQI-HUN): comparing psychiatric patients with control subjects. *Sleep & Breathing*.

[11] Buysse D. J., Reynolds C. F., Monk T. H., Berman S. R., Kupfer D. J. (1989). The Pittsburgh Sleep Quality Index: a new instrument for psychiatric practice and research. *Psychiatry Research*.

[12] Cole J. C., Motivala S. J., Buysse D. J., Oxman M. N., Levin M. J., Irwin M. R. (2006). Validation of a 3-factor scoring model for the Pittsburgh Sleep Quality Index in older adults. *Sleep*.

[13] Sohn S. I., Kim D. H., Lee M. Y., Cho Y. W. (2012). The reliability and validity of the Korean version of the Pittsburgh Sleep Quality Index. *Sleep and Breathing*.

[14] Kotronoulas G., Papadopoulou C. N., Papapetrou A., Patiraki E. (2011). Psychometric evaluation and feasibility of the Greek Pittsburgh Sleep Quality Index (GR-PSQI) in patients with cancer receiving chemotherapy. *Supportive Care in Cancer*.

[15] Tzeng J. I., Fu Y.-W., Lin C.-C. (2012). Validity and reliability of the Taiwanese version of the Pittsburgh Sleep Quality Index in cancer patients. *International Journal of Nursing Studies*.

[16] Manzar M. D., BaHammam A. S., Hameed U. A. (2018). Dimensionality of the Pittsburgh Sleep Quality Index: a systematic review. *Health and Quality of Life Outcomes*.

[17] Ho R. T. H., Fong T. C. T. (2014). Factor structure of the Chinese version of the Pittsburgh Sleep Quality Index in breast cancer patients. *Sleep Medicine*.

[18] Gomes A. A., Marques D. R., Meiavia A. M., Cunha F., Clemente V. (2018). Psychometric properties and accuracy of the European Portuguese version of the Pittsburgh Sleep Quality Index in clinical and non-clinical samples. *Sleep and Biological Rhythms*.

[19] Magee C. A., Caputi P., Iverson D. C., Huang X.-F. (2008). An investigation of the dimensionality of the Pittsburgh Sleep Quality Index in Australian adults. *Sleep and Biological Rhythms*.

[20] Nicassio P. M., Ormseth S. R., Custodio M. K., Olmstead R., Weisman M. H., Irwin M. R. (2014). Confirmatory factor analysis of the Pittsburgh Sleep Quality Index in rheumatoid arthritis patients. *Behavioral Sleep Medicine*.

[21] Babson K. A., Blonigen D. M., Boden M. T., Drescher K. D., Bonn-Miller M. O. (2012). Sleep quality among U.S. military veterans with PTSD: a factor analysis and structural model of symptoms. *Journal of Traumatic Stress*.

[22] Gelaye B., Lohsoonthorn V., Lertmeharit S. (2014). Construct validity and factor structure of the Pittsburgh Sleep Quality Index and Epworth Sleepiness Scale in a multi-national study of African, South East Asian and South American college students. *PLoS One*.

[23] Burkhalter H., Sereika S. M., Engberg S., Wirz-Justice A., Steiger J., de Geest S. (2010). Structure validity of the Pittsburgh Sleep Quality Index in renal transplant recipients: a confirmatory factor analysis. *Sleep and Biological Rhythms*.

[24] Jia Y., Chen S., Deutz N. E. P., Bukkapatnam S. T. S., Woltering S. (2019). Examining the structure validity of the Pittsburgh Sleep Quality Index. *Sleep and Biological Rhythms*.

[25] Dudysová D., Malá I., Mladá K., Saifutdinova E., Koprivova J., Sos P. (2017). Structural and construct validity of the Czech version of the Pittsburgh Sleep Quality Index in chronic insomnia. *Neuroendocrinology Letters*.

[26] Portaluppi F., Smolensky M., Touitou Y. (2010). Ethics and methods for biological rhythm research on animals and human beings. *Chronobiology International*.

[27] Zhang C., Zhang H., Zhao M. (2020). Reliability, validity, and factor structure of Pittsburgh Sleep Quality Index in community-based centenarians. *Frontiers in Psychiatry*.

[28] Fawcett T. (2006). An introduction to ROC analysis. *Pattern Recognition Letters*.

[29] Youden W. J. (1950). Index for rating diagnostic tests. *Cancer*.

[30] Cronbach L. J. (1951). Coefficient alpha and the internal structure of tests. *Psychometrika*.

[31] George D. (2003). SPSS for Windows Step by Step: A Simple Study Guide and Reference, 17.0 Update, 10/e.

[32] Field A. (2013). Discovering Statistics Using IBM SPSS Statistics.

[33] Hair J. F., Tatham R. L., Anderson R. E., Black W. (1998). *Multivariate Data Analysis*.

[34] Revelle W. (2021). How to: use the psych package for factor analysis and data reduction. http://personality-project.org/r/psych/HowTo/factor.pdf.

[35] Hooper D., Coughlan J., Mullen M. R. (2008). Structural equation modelling: guidelines for determining model fit. *The Electronic Journal of Business Research Methods*.

[36] R Development Core Team (2018). R: a language and environment for statistical computing. http://www.R-project.org.

[37] The jamovi project (2019). https://www.jamovi.org.

[38] Wickham H. (2017). tidyverse: easily install and load the "Tidyverse". https://CRAN.R-project.org/package=tidyverse.

[39] Revelle W. R. (2017). Psych: procedures for personality and psychological research. https://www.scholars.northwestern.edu/en/publications/psych-procedures-for-personality-and-psychological-research.

[40] Thiele C. (2019). An introduction to cutpointr. https://cran.r-project.org/web/packages/cutpointr/vignettes/cutpointr.html.

[41] Robin X., Turck N., Hainard A. (2011). pROC: an open-source package for R and S+ to analyze and compare ROC curves. *BMC Bioinformatics*.

[42] Cohen J. (1988). *Statistical Power Analysis for the Behavioral Sciences*.

[43] Farrahi Moghaddam J., Nakhaee N., Sheibani V., Garrusi B., Amirkafi A. (2016). Reliability and validity of the Persian version of the Pittsburgh Sleep Quality Index (PSQI-P). *Sleep and Breathing*.

[44] Raniti M. B., Waloszek J. M., Schwartz O., Allen N. B., Trinder J. (2018). Factor structure and psychometric properties of the Pittsburgh Sleep Quality Index in community-based adolescents. *Sleep*.

[45] Skouteris H., Wertheim E. H., Germano C., Paxton S. J., Milgrom J. (2009). Assessing sleep during pregnancy: a study across two time points examining the Pittsburgh Sleep Quality Index and associations with depressive symptoms. *Womens Health Issues*.

[46] Jomeen J., Martin C. R. (2007). Assessment and relationship of sleep quality to depression in early pregnancy. *Journal of Reproductive and Infant Psychology*.

[47] Tomfohr L. M., Schweizer C. A., Dimsdale J. E., Loredo J. S. (2013). Psychometric characteristics of the Pittsburgh Sleep Quality Index in English speaking non-Hispanic whites and English and Spanish speaking Hispanics of Mexican descent. *Journal of Clinical Sleep Medicine*.

[48] Li L., Sheehan C. M., Thompson M. S. (2019). Measurement invariance and sleep quality differences between men and women in the Pittsburgh Sleep Quality Index. *Journal of Clinical Sleep Medicine*.

[49] Buysse D. J., Hall M. L., Strollo P. J. (2008). Relationships between the Pittsburgh Sleep Quality Index (PSQI), Epworth Sleepiness Scale (ESS), and clinical/polysomnographic measures in a community sample. *Journal of Clinical Sleep Medicine*.

[50] Dostálová S., Šusta M., Nepožitek J. (2020). Polysomnographic findings in individuals over 50 years of age lacking subjective signs of sleep disturbance. *Česká a Slovenská Neurologie a Neurochirurgie*.

[51] Janečková D. (2014). *Cirkadiánní preference. Rozdílný život ranních ptáčat a nočních sov*.

[52] Matthews T., Danese A., Gregory A. M., Caspi A., Moffitt T. E., Arseneault L. (2017). Sleeping with one eye open: loneliness and sleep quality in young adults. *Psychological Medicine*.

[53] Franquelo-Morales P., Sánchez-López M., Notario-Pacheco B. (2018). Association between health-related quality of life, obesity, fitness, and sleep quality in young adults: the Cuenca adult study. *Behavioral Sleep Medicine*.

[54] Šmotek M., Fárková E., Manková D., Kopřivová J. (2020). Evening and night exposure to screens of media devices and its association with subjectively perceived sleep: should "light hygiene" be given more attention?. *Sleep Health*.

